# Correlation analysis between preoperative inflammation-nutrition-related indices and the risk of pancreatic fistula formation following distal pancreatectomy

**DOI:** 10.1371/journal.pone.0360418

**Published:** 2026-10-09

**Authors:** Shiwei Wu, Dongxing Zhang, Yizhang Zhu, Ning Li, Daobin Wang, Jiayue Zou, Xiaoyuan Hu, Jiawei Jin, Jun He, Xiaofeng Xue, Lei Qin

**Affiliations:** 1 Pancreatic Disease Center, The First Affiliated Hospital of Soochow University, Suzhou, Jiangsu, China; 2 Department of General Surgery, The First Affiliated Hospital of Soochow University, Suzhou, Jiangsu, China; Universitá Sapienza di Roma, ITALY

## Abstract

**Objective:**

To investigate the associations of preoperative C-reactive protein-to-albumin ratio (CAR), inflammatory burden index (IBI), and C-reactive protein-albumin-lymphocyte (CALLY) index with clinically relevant postoperative pancreatic fistula (CR-POPF) after distal pancreatectomy (DP), and to compare their predictive value, providing a reference for preoperative risk assessment.

**Methods:**

We retrospectively included 300 patients who underwent DP between June 2022 and June 2024. CR-POPF was defined according to the 2016 International Study Group of Pancreatic Surgery (ISGPS) criteria as Grade B or Grade C POPF. Multivariable logistic regression was used to evaluate the associations of CAR, IBI, and CALLY with CR-POPF, and restricted cubic splines were used to assess dose-response relationships. Predictive performance of the preoperative baseline model and models incorporating each index was compared using receiver operating characteristic (ROC) analysis with DeLong tests, calibration assessment, Brier scores, decision curve analysis, and 1,000-resample bootstrap internal validation.

**Results:**

CR-POPF occurred in 84 of 300 patients (28.0%); among these events, 81 (96.4%) were Grade B and 3 (3.6%) were Grade C. In preoperative-adjusted analyses, higher CAR (odds ratio [OR] 230.244, 95% confidence interval [CI] 45.053–1176.652; P < 0.001) and IBI (OR 1.015, 95% CI 1.006–1.024; P < 0.001) were associated with higher odds of CR-POPF, whereas higher CALLY was associated with lower odds (OR 0.979, 95% CI 0.967–0.991; P = 0.001). The baseline model had an area under the ROC curve (AUC) of 0.786 (95% CI 0.726–0.845). Adding CAR increased the AUC to 0.894 (95% CI 0.857–0.931; P < 0.001 vs baseline), compared with 0.821 for IBI (P = 0.004) and 0.827 for CALLY (P = 0.016). Bootstrap-corrected AUCs were 0.763 for the baseline model, 0.879 for baseline+CAR, 0.796 for baseline+IBI, and 0.805 for baseline+CALLY. The CAR-extended model also had the lowest Brier score (0.120) and showed the greatest net benefit across much of the examined threshold range.

**Conclusions:**

Preoperative CAR, IBI, and CALLY were associated with CR-POPF after DP. Among the three indices, CAR showed the strongest predictive performance when added to the preoperative clinical model. These findings may provide a reference for preoperative risk assessment, although external validation is needed.

## Introduction

Clinically relevant postoperative pancreatic fistula (CR-POPF) remains one of the most frequent and clinically consequential complications after distal pancreatectomy (DP), contributing to intra-abdominal infection, hemorrhage, reintervention, prolonged hospitalization, and increased health-care utilization [1–4]. Risk after DP is influenced by patient-related and pancreas-specific factors, including body habitus, pancreatic morphology, pancreatic thickness, duct diameter, tissue characteristics, and operative factors [2–5]. Nevertheless, a substantial proportion of risk may not be captured by anatomic variables alone.

Systemic inflammation, nutritional reserve, and immune status may influence tissue repair and postoperative complications. Conventional measures such as body mass index and the prognostic nutritional index primarily capture nutritional status, while composite inflammation-nutrition indices integrate multiple biological domains [6–11]. The C-reactive protein-to-albumin ratio (CAR) has been associated with postoperative complications in gastrointestinal surgery and with POPF after pancreatic surgery [10–12]. The C-reactive protein-albumin-lymphocyte (CALLY) index combines C-reactive protein (CRP), albumin, and lymphocyte count and has shown prognostic or complication-related value in several gastrointestinal malignancies [13–15]. The inflammatory burden index (IBI) combines CRP with the neutrophil-to-lymphocyte ratio and has been associated with outcomes across cancer and cardiovascular cohorts [16–19].

Accordingly, this study aimed to investigate the associations of preoperative CAR, IBI, and CALLY with CR-POPF after DP and to compare their predictive value, providing additional evidence for preoperative risk assessment.

## Materials and methods

### Study design and population

This single-center retrospective cohort study included patients who underwent DP at the Department of General Surgery and Pancreatic Disease Center of the First Affiliated Hospital of Soochow University between June 2022 and June 2024. The study was conducted in accordance with the Declaration of Helsinki and was approved by the Ethics Committee of the First Affiliated Hospital of Soochow University (Approval No. 2026596). Because of the retrospective design, the requirement for individual informed consent was waived. All data were de-identified before analysis.

### Patient selection

Inclusion criteria were: (1) DP performed during the study period; (2) no evidence of distant metastasis before surgery; (3) no neoadjuvant therapy; and (4) no history of previous pancreatic surgery. Exclusion criteria were: (1) missing key perioperative or laboratory data; (2) absence of hematologic testing within one week before surgery; or (3) CT images unsuitable for pancreas-related measurements. Preoperative computed tomography (CT) images were used to measure pancreatic neck thickness and main pancreatic duct (MPD) diameter, together with pancreatic parenchymal attenuation. A total of 300 patients met the eligibility criteria. No missing values were present for the variables included in the primary analyses.

### Clinical variables

Demographic and clinical data were obtained from the electronic medical record and anesthesia information systems. Variables included age, gender, body mass index (BMI), diabetes, pancreatic neck thickness, MPD diameter, and pancreatic parenchymal CT attenuation. Pancreatic neck thickness, MPD diameter, and CT attenuation were assessed on preoperative CT images.

### Inflammation-nutrition indices

The inflammatory markers analyzed in this study are based on calculations of peripheral blood-related indicators. Relevant indicators include neutrophil count (×10^9^/L), lymphocyte count (×10^9^/L), albumin level (g/L), and C-reactive protein (CRP) level (mg/L). The calculation formulas for each inflammation-nutrition index are as follows:


$$\begin{array}{c} \text{CAR}=\text{C}-\text{reactive protein level}/\text{Albumin level} \end{array}$$

$$\begin{array}{c} \text{IBI}=(\text{C}-\text{reactive protein level}\times \text{Neutrophil count})/\text{Lymphocyte count} \end{array}$$

$$\begin{array}{c} \text{CALLY}=(\text{Albumin}\times \text{Lymphocyte})/(\text{CRP}\times \text{10}) \end{array}$$

### Definition and adjudication of CR-POPF

Postoperative pancreatic fistula (POPF) was classified according to the 2016 International Study Group of Pancreatic Surgery (ISGPS) update [1]. The primary outcome was CR-POPF, defined as Grade B or Grade C POPF. A biochemical leak was not classified as POPF under the 2016 ISGPS definition. Grade B POPF was defined by a clinically relevant change in postoperative management, including drains left in place for >3 weeks or repositioned through endoscopic or percutaneous procedures, without Grade C criteria. Grade C POPF was defined by POPF-related reoperation, organ failure, and/or death.

### Statistical analysis

Normally distributed continuous variables are presented as mean ± standard deviation and were compared using the independent-samples t test. Skewed continuous variables are presented as median [interquartile range] and were compared using the Mann-Whitney U test. Categorical variables are presented as n (%) and were compared using the chi-square test or Fisher exact test, as appropriate. For each inflammation-nutrition index, crude and preoperative-adjusted logistic regression models were fitted. The adjusted model included age, gender, BMI, diabetes, pancreatic neck thickness, MPD diameter, and pancreatic parenchymal CT attenuation. Each index was evaluated as a continuous variable and by quartiles; P for trend was calculated by assigning each quartile its median value and entering that value as a continuous term. Because CAR, IBI, and CALLY all contain CRP, the three indices were evaluated in separate multivariable models. Spearman correlation coefficients were calculated to assess correlations among the three indices. Restricted cubic spline (RCS) analyses used four knots placed at the 5th, 35th, 65th, and 95th percentiles, with the median value of each index as the reference. Overall and nonlinear associations were tested. Exploratory subgroup analyses were conducted by gender, diabetes, age (<65 vs ≥ 65 years), and BMI (<25 vs ≥ 25 kg/m²). Interaction terms were assessed using likelihood-ratio tests. A preoperative baseline model was compared with models adding CAR, IBI, or CALLY. Discrimination was quantified by the area under the receiver operating characteristic (ROC) curve (AUC) with 95% confidence intervals and compared using paired DeLong tests. Likelihood-ratio tests were used to compare model fit. Calibration was assessed using bootstrap calibration curves, and overall prediction error was summarized with the Brier score. Decision curve analysis was used to evaluate net benefit across threshold probabilities of 0.01–0.50. Internal validation was performed using 1,000 bootstrap resamples to estimate optimism-corrected AUC and Brier scores. As a supplementary benchmark, the preoperative Distal Pancreatectomy Fistula Risk Score (D-FRS) was calculated using pancreatic neck thickness and MPD diameter according to the published model [5], and its discrimination was assessed using ROC analysis. Sensitivity analyses used natural-log-transformed and standardized versions of CAR, IBI, and CALLY, with odds ratios expressed per 1-SD increase. All tests were two-sided, with P < 0.05 considered statistically significant. Analyses were performed using R version 4.4.1.

## Results

### Baseline characteristics

A total of 300 patients were included, of whom 84 (28.0%) developed CR-POPF and 216 (72.0%) did not. Among the 84 CR-POPF events, 81 (96.4%) were Grade B and 3 (3.6%) were Grade C. Compared with patients without CR-POPF, patients with CR-POPF had a higher proportion of women (52.4% vs 34.3%, P = 0.006), greater pancreatic neck thickness (13.72 vs 11.68 mm, P < 0.001), and lower pancreatic CT attenuation (36.44 vs 43.20, P < 0.001). CAR and IBI were higher, whereas CALLY was lower, in the CR-POPF group (all P < 0.001). Other baseline characteristics are summarized in Table 1.

**Table 1:** Baseline characteristics of study participants grouped by CR-POPF status.

Variable	Level	Overall (n = 300)	Non-CR-POPF (n = 216)	CR-POPF (n = 84)	P
**Age (mean (SD))**		57.63 (15.17)	56.68 (15.55)	60.08 (13.93)	0.081
**Gender, n (%)**	Male	182 (60.7)	142 (65.7)	40 (47.6)	0.006
	Female	118 (39.3)	74 (34.3)	44 (52.4)	
**BMI (mean (SD))**		23.33 (3.43)	23.35 (3.54)	23.30 (3.14)	0.909
**Diabetes, n (%)**	No	232 (77.3)	168 (77.8)	64 (76.2)	0.888
	Yes	68 (22.7)	48 (22.2)	20 (23.8)	
**Pancreatic neck thickness, mm (mean (SD))**		12.25 (3.42)	11.68 (3.00)	13.72 (3.98)	<0.001
**Main pancreatic duct diameter, mm (mean (SD))**		1.84 (0.89)	1.79 (0.89)	1.98 (0.88)	0.101
**CT (Pancreas) (mean (SD))**		41.31 (8.49)	43.20 (7.86)	36.44 (8.17)	<0.001
**CAR (median [IQR])**		0.10 [0.02, 0.34]	0.05 [0.02, 0.16]	0.36 [0.27, 0.50]	<0.001
**IBI (median [IQR])**		7.77 [1.72, 29.93]	3.66 [1.14, 14.14]	29.40 [17.13, 45.41]	<0.001
**CALLY (median [IQR])**		5.37 [1.71, 32.16]	10.30 [2.20, 53.58]	3.30 [0.22, 5.69]	<0.001

Continuous variables with skewed distributions are presented as median [interquartile range] where specified; categorical variables are presented as n (%). Abbreviations: BMI, body mass index; CT, computed tomography; CAR, C-reactive protein-to-albumin ratio; IBI, inflammatory burden index; CALLY, C-reactive protein-albumin-lymphocyte index; SD, standard deviation; IQR, interquartile range; CR-POPF, clinically relevant postoperative pancreatic fistula.

### Associations of CAR, IBI, and CALLY with CR-POPF

In continuous-variable analyses, higher CAR and IBI were associated with increased odds of CR-POPF, whereas higher CALLY was associated with decreased odds. After adjustment for the preoperative clinical covariates, the adjusted odds ratios (ORs) were 230.244 (95% confidence interval [CI] 45.053–1176.652; P < 0.001) for CAR, 1.015 (95% CI 1.006–1.024; P < 0.001) for IBI, and 0.979 (95% CI 0.967–0.991; P = 0.001) for CALLY. In quartile analyses, the highest quartile of CAR was associated with higher odds of CR-POPF than Q1 (adjusted OR 36.477, 95% CI 10.644–125.004; P < 0.001), as was the highest quartile of IBI (adjusted OR 29.030, 95% CI 7.423–113.532; P < 0.001). In contrast, the highest CALLY quartile was associated with lower odds (adjusted OR 0.220, 95% CI 0.076–0.641; P = 0.006). P for trend was < 0.001 for all three indices (Table 2).

**Table 2:** Association between preoperative inflammation-nutrition indices and CR-POPF.

Index	Exposure	Crude Model	P	Preoperative-adjusted Model	P
CAR	Continuous	304.605 (66.398-1397.405)	<0.001	230.244 (45.053-1176.652)	<0.001
	Q1	Ref		Ref	
	Q2	0.486 (0.086-2.739)	0.414	0.304 (0.035-2.646)	0.281
	Q3	10.574 (3.483-32.106)	<0.001	13.885 (4.049-47.616)	<0.001
	Q4	35.500 (11.632-108.346)	<0.001	36.477 (10.644-125.004)	<0.001
	P for trend		<0.001		<0.001
IBI	Continuous	1.016 (1.008-1.025)	<0.001	1.015 (1.006-1.024)	<0.001
	Q1	Ref		Ref	
	Q2	2.087 (0.502-8.673)	0.311	1.789 (0.366-8.748)	0.473
	Q3	21.000 (6.074-72.608)	<0.001	29.679 (7.453-118.189)	<0.001
	Q4	27.429 (7.933-94.835)	<0.001	29.030 (7.423-113.532)	<0.001
	P for trend		<0.001		<0.001
CALLY	Continuous	0.975 (0.962-0.988)	<0.001	0.979 (0.967-0.991)	0.001
	Q1	Ref		Ref	
	Q2	1.397 (0.725-2.691)	0.318	2.176 (1.006-4.707)	0.048
	Q3	0.561 (0.276-1.141)	0.111	0.885 (0.380-2.064)	0.778
	Q4	0.155 (0.059-0.403)	<0.001	0.220 (0.076-0.641)	0.006
	P for trend		<0.001		<0.001

Values are odds ratios (ORs) with 95% confidence intervals (CIs). The preoperative-adjusted model includes age, gender, BMI, diabetes, pancreatic neck thickness, main pancreatic duct diameter, and pancreatic CT attenuation. P for trend was calculated by entering the median value of each quartile as a continuous variable. Abbreviations: BMI, body mass index; CT, computed tomography; CAR, C-reactive protein-to-albumin ratio; IBI, inflammatory burden index; CALLY, C-reactive protein-albumin-lymphocyte index; CR-POPF, clinically relevant postoperative pancreatic fistula.

Spearman correlation analysis showed a strong positive correlation between CAR and IBI (rho = 0.941), while CALLY was inversely correlated with CAR (rho = −0.559) and IBI (rho = −0.557) (Table 3).

**Table 3:** Spearman correlation coefficients among CAR, IBI, and CALLY.

Index	CAR	IBI	CALLY
CAR	1.000	0.941	−0.559
IBI	0.941	1.000	−0.557
CALLY	−0.559	−0.557	1.000

Abbreviations: CAR, C-reactive protein-to-albumin ratio; IBI, inflammatory burden index; CALLY, C-reactive protein-albumin-lymphocyte index.

### Restricted cubic splines and exploratory subgroup analyses

RCS analyses showed significant overall associations for CAR, IBI, and CALLY (all P overall <0.001). There was evidence of nonlinearity for CAR and IBI (both P nonlinear <0.001), whereas the association for CALLY was not significantly nonlinear (P nonlinear = 0.083) (Fig 1).

**Fig 1:**
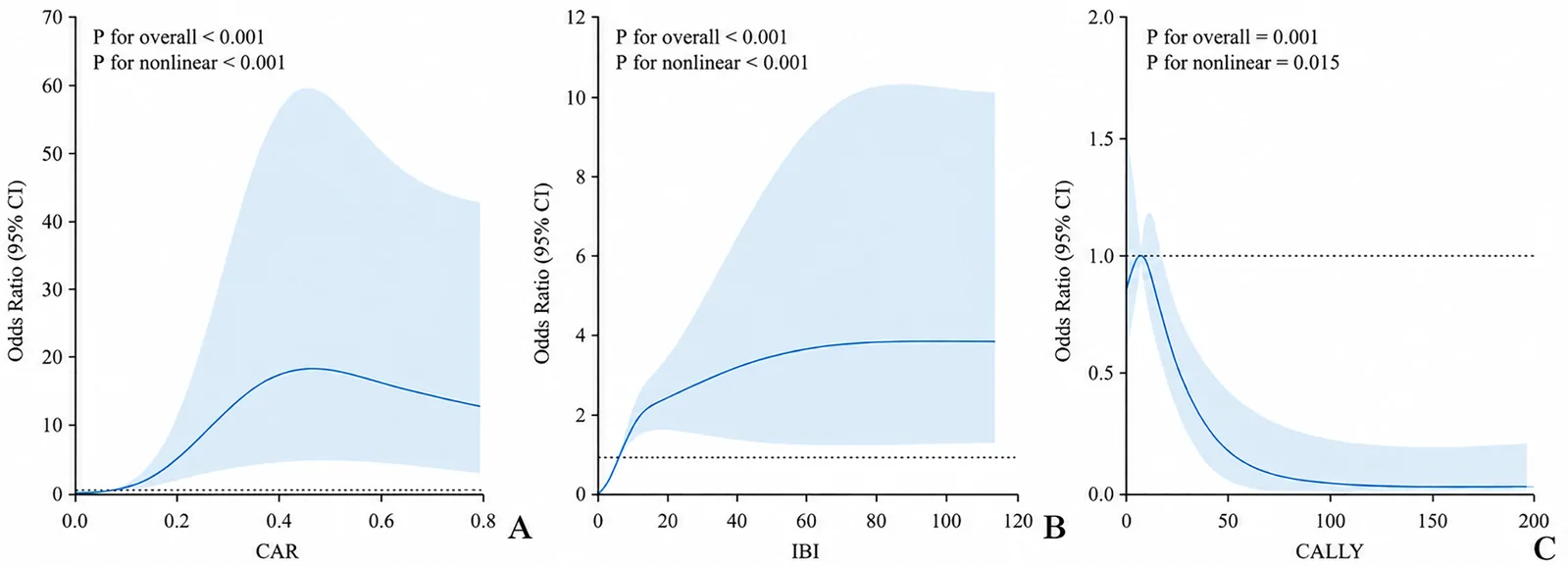
Restricted cubic spline (RCS) analyses. (A) CAR; (B) IBI; (C) CALLY. Models were adjusted for age, gender, BMI, diabetes, pancreatic neck thickness, MPD diameter, and pancreatic CT attenuation. Four knots were placed at the 5th, 35th, 65th, and 95th percentiles, with the median as the reference. Abbreviations: RCS, restricted cubic spline; BMI, body mass index; MPD, main pancreatic duct; CT, computed tomography; CAR, C-reactive protein-to-albumin ratio; IBI, inflammatory burden index; CALLY, C-reactive protein-albumin-lymphocyte index.

Exploratory subgroup analyses are shown in Fig 2. Interaction tests suggested heterogeneity for CAR by diabetes status (P interaction = 0.002) and for IBI by age (P interaction<0.001) and BMI (P interaction = 0.025). No significant interaction was detected for CALLY.

**Fig 2:**
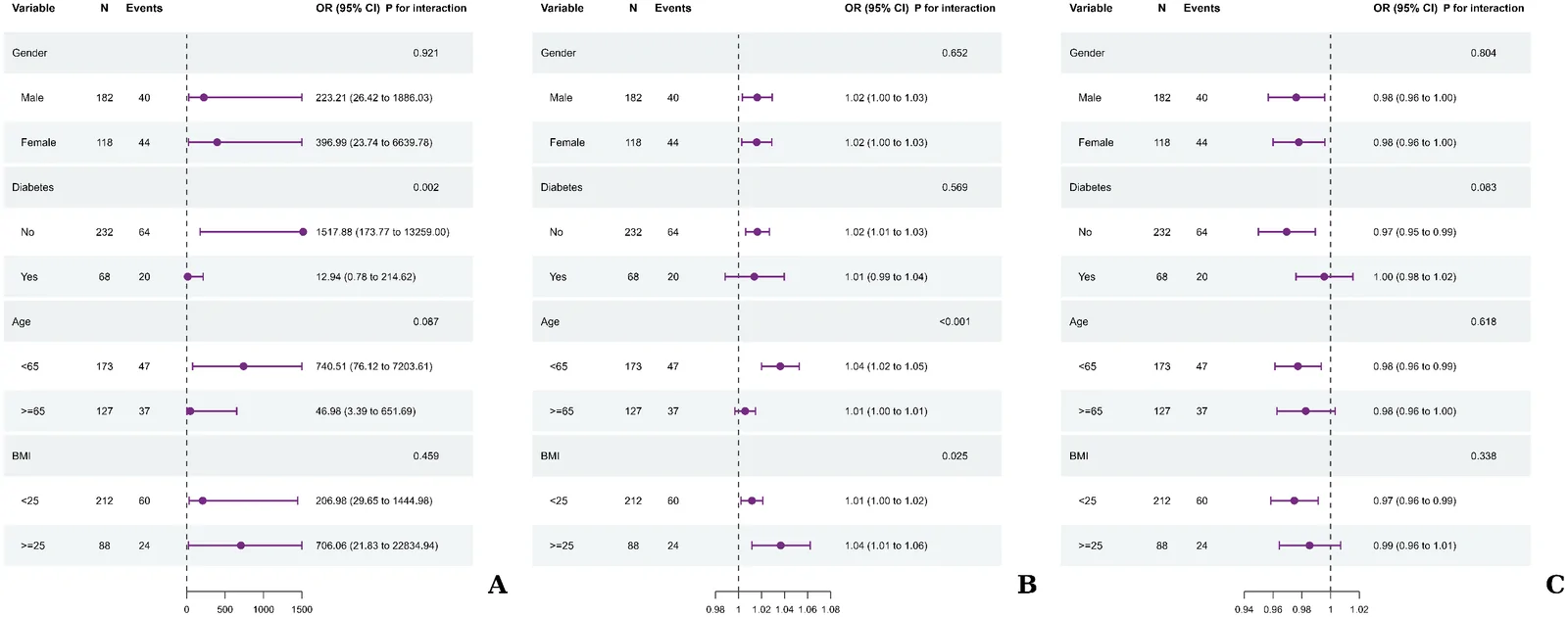
Exploratory subgroup analyses. (A) CAR, (B) IBI, and (C) CALLY. Subgroup findings are exploratory; P for interaction was derived from likelihood-ratio tests. Abbreviations: CAR, C-reactive protein-to-albumin ratio; IBI, inflammatory burden index; CALLY, C-reactive protein-albumin-lymphocyte index.

### Predictive performance and internal validation

The preoperative baseline model showed an AUC of 0.786 (95% CI 0.726–0.845). Adding CAR increased the AUC to 0.894 (95% CI 0.857–0.931), with a significant improvement compared with the baseline model by the DeLong test (P < 0.001). The corresponding AUCs were 0.821 (95% CI 0.766–0.875) for baseline+IBI and 0.827 (95% CI 0.776–0.879) for baseline+CALLY, which were also significantly higher than that of the baseline model (DeLong P = 0.004 and P = 0.016, respectively). Likelihood-ratio tests showed significant improvement in model fit after addition of each index (all P < 0.001). The baseline+CAR model also had the lowest Brier score (0.120), compared with 0.153 for the baseline model, 0.144 for baseline+IBI, and 0.141 for baseline+CALLY. Bootstrap calibration curves showed that predicted and observed probabilities were generally consistent across the models, with the curves remaining reasonably close to the ideal line. After 1,000 bootstrap resamples, the optimism-corrected AUCs were 0.763 for the baseline model, 0.879 for baseline+CAR, 0.796 for baseline+IBI, and 0.805 for baseline+CALLY; the corresponding optimism-corrected Brier scores were 0.163, 0.130, 0.157, and 0.151. Decision curve analysis further showed that the baseline+CAR model provided the greatest net benefit across much of the examined threshold-probability range (Table 4 and Fig 3). As a supplementary benchmark analysis, the preoperative D-FRS showed modest discrimination for CR-POPF in the present cohort, with an AUC of 0.649 (95% CI 0.578–0.719) (S1 Fig).

**Table 4:** Predictive performance and internal validation of the preoperative prediction models.

Model	AUC (95% CI)	Corrected AUC	DeLong test P	Likelihood-ratio test P	Brier score	Corrected Brier
Baseline	0.786 (0.726-0.845)	0.763			0.153	0.163
Baseline + CAR	0.894 (0.857-0.931)	0.879	<0.001	<0.001	0.120	0.130
Baseline + IBI	0.821 (0.766-0.875)	0.796	0.004	<0.001	0.144	0.157
Baseline + CALLY	0.827 (0.776-0.879)	0.805	0.016	<0.001	0.141	0.151

AUCs were compared with the baseline model using paired DeLong tests. Likelihood-ratio tests comparing each extended model with the baseline model were significant. Internal validation used 1,000 bootstrap resamples. Abbreviations: AUC, area under the receiver operating characteristic curve; CI, confidence interval; CAR, C-reactive protein-to-albumin ratio; IBI, inflammatory burden index; CALLY, C-reactive protein-albumin-lymphocyte index.

**Fig 3:**
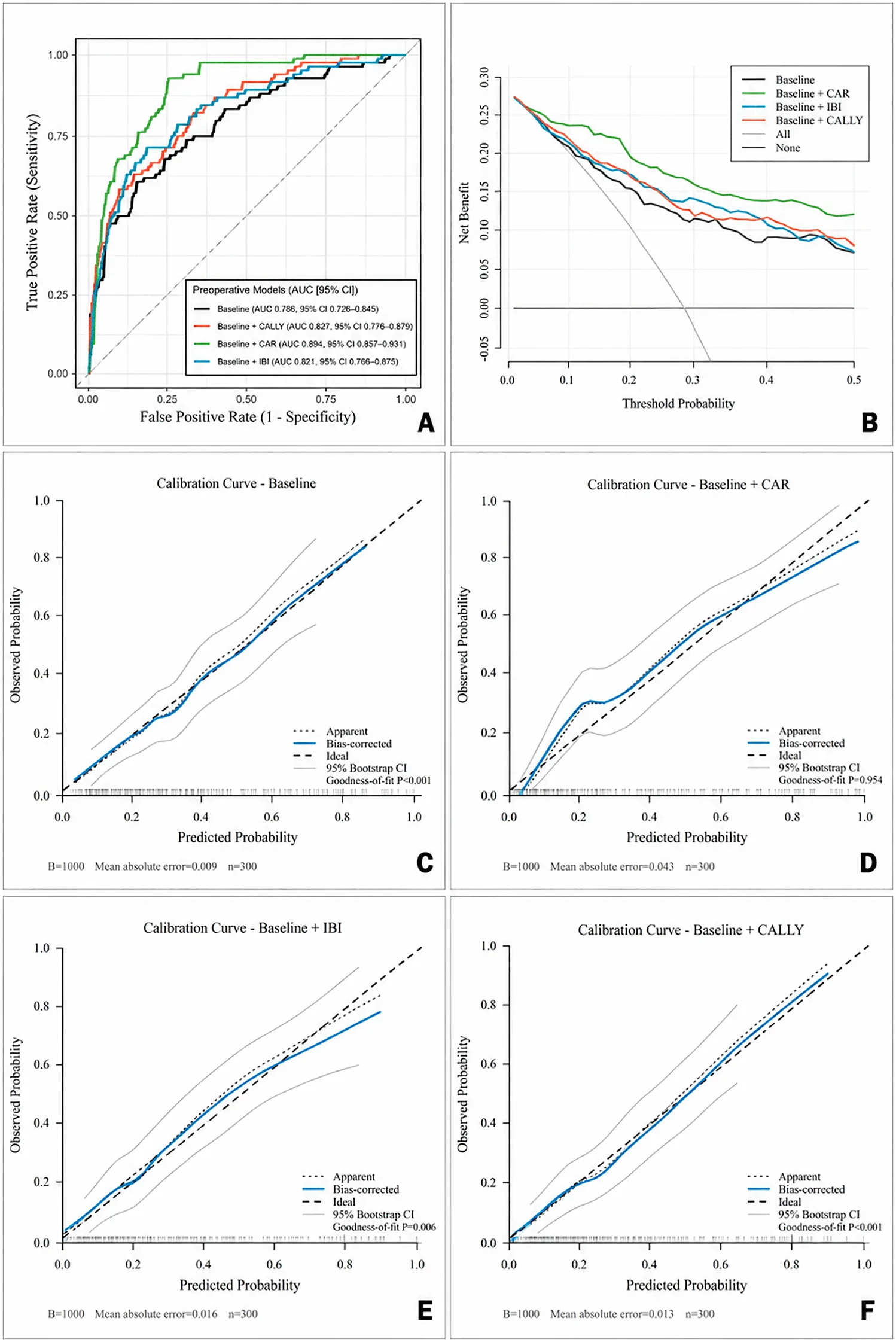
Predictive performance and internal validation of the baseline model and models extended with CAR, IBI, or CALLY. (A) ROC curves comparing the baseline preoperative model with models additionally incorporating CAR, IBI, or CALLY. (B) Decision curve analysis comparing the net clinical benefit of the baseline model and the three extended models across threshold probabilities from 0.01 to 0.50. (C) Bootstrap calibration curve for the baseline model. (D) Bootstrap calibration curve for the baseline + CAR model. (E) Bootstrap calibration curve for the baseline + IBI model. (F) Bootstrap calibration curve for the baseline + CALLY model. Calibration curves were generated using 1,000 bootstrap resamples; the diagonal line represents perfect calibration. Abbreviations: ROC, receiver operating characteristic; AUC, area under the ROC curve; CAR, C-reactive protein-to-albumin ratio; IBI, inflammatory burden index; CALLY, C-reactive protein-albumin-lymphocyte index.

### Sensitivity analyses

Results were consistent when the indices were natural-log transformed and standardized. Per 1-SD increase in the log-transformed index, the preoperative-adjusted OR was 7.089 (95% CI 4.079–12.320; P < 0.001) for CAR, 4.514 (95% CI 2.867–7.108; P < 0.001) for IBI, and 0.512 (95% CI 0.370–0.708; P < 0.001) for CALLY (Table 5).

**Table 5:** Sensitivity analysis.

Index	Model	OR (95% CI)	P
CAR	Crude	7.209 (4.317-12.039)	<0.001
	Preoperative-adjusted	7.089 (4.079-12.320)	<0.001
IBI	Crude	4.194 (2.819-6.239)	<0.001
	Preoperative-adjusted	4.514 (2.867-7.108)	<0.001
CALLY	Crude	0.447 (0.336-0.595)	<0.001
	Preoperative-adjusted	0.512 (0.370-0.708)	<0.001

ORs are expressed per 1-SD increase in the natural-log-transformed index. Abbreviations: OR, odds ratio; CI, confidence interval; SD, standard deviation; CAR, C-reactive protein-to-albumin ratio; IBI, inflammatory burden index; CALLY, C-reactive protein-albumin-lymphocyte index.

## Discussion

In this retrospective cohort of patients undergoing DP, higher CAR and IBI were associated with greater odds of CR-POPF, whereas higher CALLY was associated with lower odds. When each index was incorporated into the same preoperative clinical model, CAR showed the largest improvement in discrimination. The direction of these associations remained consistent in sensitivity analyses, and bootstrap validation supported the relative performance of the CAR model.

Pancreatic fistula risk after DP is multifactorial. Previous studies have identified patient-related, pancreas-specific, and operative risk factors [2–5]. In the present analysis, pancreatic neck thickness, MPD diameter, and pancreatic CT attenuation were considered together with basic clinical variables so that the inflammation-nutrition indices could be evaluated in the context of relevant preoperative characteristics.

The observed associations are biologically plausible but should not be interpreted causally. Systemic inflammation may be linked to immune dysregulation, impaired tissue repair, and postoperative infectious complications, while low albumin and lymphocyte-related measures may reflect reduced nutritional or immune reserve. Previous studies have linked the neutrophil-to-lymphocyte ratio (NLR) to fistula healing after DP [20], postoperative inflammatory scores to POPF after pancreatic resection [21], and preoperative CAR to POPF risk in pancreatic surgery [12,22]. Our results extend these observations by evaluating CAR, IBI, and CALLY within the same DP cohort and by quantifying their incremental contribution beyond a common preoperative clinical model.

Bootstrap internal validation showed limited optimism, and the CAR model retained the highest optimism-corrected AUC. Its Brier score was also the lowest among the compared models, while decision curve analysis suggested potential clinical utility across a range of threshold probabilities. These findings remain internally derived and require confirmation in external cohorts. As a supplementary benchmark, the preoperative D-FRS showed modest discrimination in the present cohort (AUC 0.649, 95% CI 0.578–0.719). This analysis was used as a supplementary benchmark and was not incorporated into the primary incremental model comparison.

Exploratory subgroup analyses suggested possible effect modification for CAR by diabetes status and for IBI by age and BMI. Because these analyses involved smaller strata and multiple comparisons, the findings should be regarded as hypothesis-generating and require independent confirmation.

Several limitations should be acknowledged. First, the single-center retrospective design may be affected by selection bias and residual confounding, and causal inference is not possible. Second, pancreatic texture and detailed pancreatic stump management were not available in the analytic dataset. Third, the inflammation-nutrition indices were based on a single preoperative measurement. In addition, CAR, IBI, and CALLY all contain CRP and therefore represent partially overlapping inflammatory information. Finally, the models underwent internal bootstrap validation but have not yet been externally validated.

## Conclusions

Preoperative CAR, IBI, and CALLY were associated with CR-POPF after DP. CAR showed the strongest predictive performance among the three indices when incorporated into the preoperative clinical model. These indices may provide additional information for preoperative risk assessment, but prospective multicenter validation is needed before clinical application.

## Supporting information

S1 Fig Receiver operating characteristic curve of the preoperative Distal Pancreatectomy Fistula Risk Score (D-FRS) for predicting CR-POPF. The preoperative D-FRS was calculated using pancreatic neck thickness and main pancreatic duct diameter obtained from preoperative CT. The AUC was 0.649 (95% CI 0.578–0.719). Abbreviations: D-FRS, Distal Pancreatectomy Fistula Risk Score; CR-POPF, clinically relevant postoperative pancreatic fistula; CT, computed tomography; AUC, area under the receiver operating characteristic curve; CI, confidence interval.
(TIF)
